# *Wolbachia* Surface Protein (*wsp*) Gene Sequencing of Strains A and B in Native *Aedes albopictus* of Mérida, Yucatán

**DOI:** 10.3390/biology14101399

**Published:** 2025-10-13

**Authors:** Henry Puerta-Guardo, Yamili Contreras-Perera, Silvia Perez-Carrillo, Azael Che-Mendoza, Karina Jacqueline Ciau-Carrillo, Manuel Parra-Cardeña, Iram Rodriguez-Sanchez, Mayra A. Gomez-Govea, Anuar Medina-Barreiro, Guadalupe Ayora-Talavera, Norma Pavia-Ruz, Abdiel Martin-Park, Pablo Manrique-Saide

**Affiliations:** 1 Centro de Investigaciones Regionales, Dr. Hideyo Noguchi, Universidad Autonoma de Yucatán (UADY), Mérida C.P. 97315, Yucatán, Mexico; jciauca@gmail.com (K.J.C.-C.); manuelparra9789@gmail.com (M.P.-C.); lupitaayoratalaver49@gmail.com (G.A.-T.); norma_pavia_ruz@hotmail.com (N.P.-R.); 2 Unidad Colaborativa de Bioensayos Entomológicos (UCBE) y del Laboratorio de Control Biológico (LCB) para *Ae. aegypti*, Universidad Autónoma de Yucatán (UADY), Campus de Ciencias Biológicas y Agropecuarias, Km. 15.5 Carr. Mérida-Xmatkuil s.n., Mérida C.P. 97315, Yucatán, Mexico; yamjaz_85@hotmail.com (Y.C.-P.); or silmaggui@gmail.com (S.P.-C.); achemendoza@gmail.com (A.C.-M.); anuar.medina@correo.uady.mx (A.M.-B.); pablo_manrique2000@hotmail.com (P.M.-S.); 3 Maladies Infectieuses et Vecteurs: Ecologie, Génétique, Evolution et Contrôle (MIVEGEC), Research Institute for Development (IRD), Université Montpellier, 34090 Montpellier, France; 4 Laboratorio de Fisiología Molecular y Estructural, Facultad de Ciencias Biológicas, Universidad Autónoma de Nuevo León, San Nicolás de los Garza C.P. 66455, Nuevo León, Mexico; iramrodriguez@gmail.com (I.R.-S.); mayra.gomezgv@uanl.edu.mx (M.A.G.-G.)

**Keywords:** *Aedes albopictus*, *Wolbachia*, supergroups A and B, *wsp* gene sequencing, Yucatán, México

## Abstract

*Aedes* (*Stegomyia*) *albopictus* (Skuse), a mosquito vector with increasing capacity to transmit human arboviral infections worldwide, naturally harbors the intracellular bacterium *Wolbachia* spp. This relationship has been observed in native mosquito populations of *Ae. albopictus* around the world and, more recently, in Mérida, Yucatán, México (abbreviated hereafter as MID). This study provides additional evidence confirming the presence of *Wolbachia* A (wAlbA) and B (wAlbB) strains in field-collected *Ae. albopictus* of Mérida, based on *wsp* gene DNA sequencing analyses of 14 positive PCR samples. Maximum likelihood (ML) analyses of the *wsp* gene showed high nucleotide sequence homology between *Wolbachia* from Merida and the globally reported strains A and B, suggesting that these two *Wolbachia* strains have remained stable in *Ae. albopictus* over time, regardless of the geographical area. To the best of our knowledge, this is the first report of *Wolbachia* gene sequencing in native populations of *Ae. albopictus* in Yucatán, Mexico. Currently many *Aedes* mosquitoes (e.g., *Ae. aegypti*) harboring intracellular *Wolbachia* B bacteria are being released as part of arbovirus and mosquito control programs in Yucatan and globally. Understanding the presence and stability of *Wolbachia* strains across different *Aedes* host backgrounds is crucial to ensuring the long-term effectiveness of biological interventions aimed at controlling mosquito populations and arbovirus transmission in endemic areas.

## Simple Summary

The mosquito *Aedes* (*Stegomyia*) *albopictus* is an invasive species that has spread across nearly all continents and is increasingly associated with the transmission of *Aedes*-borne human viruses (ABVs), such as dengue (DENV), chikungunya (CHIKV), yellow fever (YFV), and Zika (ZIKV). *Wolbachia*-based strategies are currently among the most innovative alternative biological methods to control vector populations. *Wolbachia* is an endosymbiotic bacterium known to interfere with the reproductive mechanisms of vectors and, consequently, with virus replication. Previous studies have demonstrated that *Ae. albopictus* is naturally associated with two *Wolbachia* strains, A and B. This study provides additional *wsp* gene DNA sequencing evidence confirming that native populations of *Ae. albopictus* in Yucatán—an arbovirus-endemic region—naturally harbor both *Wolbachia* strains A and B. These findings present an opportunity to strategically plan future surveillance and control programs targeting *Ae. albopictus* through *Wolbachia* strain B-based approaches, with the potential to lower the burden of arbovirus diseases in human populations.

## 1. Introduction

*Wolbachia* is a diverse genus of obligate intracellular Gram-negative α-Proteobacteria (order: Rickettsiales) that can be maternally transmitted. *Wolbachia* have been suggested to infect at least 20% of arthropod species, where an estimated 40–65% are insects, among which 28% are mosquito species, as well as filarial nematodes of mammals and plants [1,2]. All *Wolbachia* strains are classified as a single species, *Wolbachia pipientis*, phylogenetically divided into 16 clades named supergroups, denoted from A to Q, mainly based on multilocus sequence typing (MLST) analysis as well as on amino acid sequence analyses of the *Wolbachia* Surface Protein (*wsp*) [3,4,5].

*Wolbachia* displays tropism for somatic and reproductive tissues of arthropod hosts, where they can manipulate their reproductive abilities by inducing cytoplasmic incompatibility (CI) [6,7]. CI confers a reproductive advantage to *Wolbachia*-infected females over uninfected females, with subsequent persistence and spread of *Wolbachia* in mosquito populations [8]. This reproductive manipulation has attracted significant interest as it plays a critical role in host biology, ecology, and evolution, as well as in the development of a symbiont-based *Wolbachia*-based method termed the Incompatible Insect Technique (IIT) for the control of insects of medical and agricultural importance [9].

Additionally, *Wolbachia* endosymbionts have been evaluated for their ability to suppress *Aedes*-borne human diseases (ABDs), including dengue, Zika, and chikungunya [10,11,12,13]. Along with *Aedes* (*Stegomyia*) *aegypti*, considered the main vector for many of these arboviral diseases, the commonly known Asian Tiger mosquito *Ae. albopictus* is a competent vector of ABDs [14,15]. *Ae. albopictus* is an aggressive biting mosquito that has invaded and colonized many countries in the Americas, Europe, Africa, and the Pacific [16,17,18].

Unlike *Ae. aegypti*, *Ae. albopictus* has been shown to naturally harbor *Wolbachia*, primarily strains from supergroups A and B [19,20,21,22]. Worldwide, although various programs using the release of *Wolbachia*-infected mosquitoes to suppress/replace natural mosquito populations have been carried out in several countries, including Australia, Brazil, Colombia, Mexico, Indonesia, and Vietnam [23,24,25], in the Americas, there are a handful of studies describing the presence of *Wolbachia* in wild populations of *Aedes* mosquitoes, including *Ae. albopictus* [26,27,28,29,30]. *Wolbachia* strains and their subgroups present in *Ae. albopictus* can serve as markers for inferring the geographic origin of mosquito populations, since both the *Aedes* spp. reservoir and bacteria strains often have specific geographic distributions, reflecting dispersal patterns and evolutionary retreats [1,2,5,19,20]. Therefore, analyzing the distribution of these strains can identify phylogenetic relationships and trace the biogeographic history of *Ae. albopictus* populations in different regions.

The *wsp* gene is commonly used as a marker for screening *Wolbachia* presence/infection, as well as for strain typing and phylogenetic analyses [3,4,31]. In this study, we obtained nucleotide sequences of the *wsp* gene obtained from *Ae. albopictus* that were positive for *Wolbachia* strains A and B according to PCR, and conducted DNA sequencing analyses to confirm the presence of these two strains and their relationship to other *Wolbachia* strains previously reported in *Ae. albopictus* and other mosquito species worldwide. In Mexico, the Ministry of Health has incorporated a *Wolbachia*-based strategy for the replacement of *Aedes aegypti* populations as part of the national dengue and arboviral disease control plan. It is anticipated that the use of *Wolbachia*-infected *Aedes* mosquitoes, including *Ae. aegypti* and *Ae. albopictus*, will expand in the coming years, not only in Mexico but also in other Latin American countries and globally. Therefore, it is essential to expand the understanding of the *Wolbachia* bacteria circulating in local mosquito populations, as this will facilitate the design of future *Wolbachia*-based intervention strategies for the control and management of mosquito-borne diseases in endemic areas.

## 2. Materials and Methods

### 2.1. Mosquito Sample Collection and Identification

In 2019 from April to December, a total of 45 *Ae. albopictus* adult mosquitoes were collected in distinct suburban areas of Mérida, Yucatán (abbreviated hereafter as MID) using outdoor BG-sentinel traps, as previously described in [30,32]. The identity of all mosquitoes was established based on standard taxonomic keys [33]. Field collections were performed every week from April to December of 2019 in three suburban areas located at the periphery of the city of Merida in the Peninsula of Yucatan: San Pedro Chimay (20°51′55″ N 89°34′46″ O), Hacienda Tahdzibichen (20°53′06″ N 89°35′52″ O), and Tekik de Regil (hereafter Tekik; 20°48′59″ N 89°33′39″ O). The average altitude of the localities is 9 m above sea level, with annual average temperatures ranging from 26 °C to 27 °C (36 °C max–18 °C min), relative humidity of 70–75%, and two distinct climate phases during the year: a rainy season, from May/June to October, with a rainfall of 882.5 mm, and a dry season, from November to April, with rainfall of 167.9 mm. The sociodemographic features of these localities include an average of 1200 inhabitants per locality with an average of 6 households and 31 inhabitants per hectare. These areas share similar urban and ecological characteristics, such as type of housing, and share large vegetated backyards with vegetation (coverage > 60%) [30].

### 2.2. DNA Extraction and Wolbachia PCR Amplification

Total genomic DNA was extracted from individual mosquitoes (n = 19, female = 11; male = 8) using a Blood and Tissue DNeasy Kit© (Qiagen, Hilden, Germany) according to the manufacturer’s instructions, with some in house modifications, as previously described in [30]. Total extracted DNA was eluted using nuclease-free water and quantified by spectrophotometry using a Nanodrop One Microvolume UV-Vis system (Thermo Scientific, Madison, WI, USA). PCR amplification of the *Wolbachia* DNA genome was performed in PCR reactions of 15 µL containing a DNA template (200 ng per reaction), PCR buffer (10×), MgCl_2_ (50 mM), dNTPs mix (10 mM), Taq DNA polymerase (5 U/µL), RNAse/DNAse-free water, and forward and reverse primers (10 µM) to amplify the DNA genome from *Wolbachia* strains A (*w*AlbA) and B (*w*AlbB) as follow: forward primers named 328F (5′-CCAGCAGATACTATTGCG-3′) and 183F (5′-AAGGAACCGAAGTTCATG-3′) for the wAlbA and wAlbB strains, respectively. For both strains, the only reverse primer 691R (5′-AAAAATTAAACGCTACTCCA-3′) was used as previously described in [30,34,35]. The PCR amplification program was performed using the following parameters: initial denaturation at 95 °C for 5 min; 40 cycles of denaturation at 95 °C for 1 min, Tm annealing at 55 °C for 1 min and extension at 72 °C for 1 min; and final extension at 72 °C for 3 min. All PCR amplifications were performed using a Mastercycler epgradient S PCR thermal cycler (Eppendorf AG, Hamburg, Germany). The presence of *Wolbachia* in *Ae. albopictus* was screened based on the amplification of 300–600 bp fragments. PCR amplicons were separated on agarose gel at 1.5% and visualized using a ChemiDoc^TM^ MP Imaging system with Image Lab software V 2.0.1 9 (Bio-Rad Laboratories, Hercules, CA, USA).

### 2.3. Wolbachia wsp Gene DNA Sequencing Analyses

Double-positive PCR amplicons [n = 30; *w*albA(+) = 15; *w*AlbB(+) = 15] were enzymatically cleaned up using ExoSAP-IT™ PCR Product Cleanup Reagent (Thermo Scientific) and then submitted for standard Sanger DNA sequencing (500 ng per sample) to PSOMAGEN, Inc. (formerly Macrogen Corp., Rockville, MD, USA), using the two sets of forward primers 328F and 182F, and one reverse primer, 691R (5 pmol/μL), as described above. A total of 60 linear DNA sequences were obtained from 19 analyzed samples using three sets of primers. The obtained partial sequences were processed for editing and analyzed using Geneious software version 6.1 (Biomatters. available at http://www.geneious.com accessed on 1 June 2023). Alignment between reference sequences and sequences in the study group was edited and aligned using the MUSCLE tool (v5.0-5.3, https://drive5.com/software.html accessed on 1 June 2023) supported by Geneious software version 6.1. To do so, a pairwise MUSCLE alignment analysis of all raw experimental nucleotide sequences [328F vs. 691R (n = 30), and 182F vs. 691R (n = 30), (n = 60 total sequences)] was performed against a group of reference sequences (n = 100), obtained from the GenBank dataset of the National Center for Biotechnology Information (NCBI), for the *wsp* gene of the *w*AlbA and *w*AlbB strains. This process allowed us to remove poorly aligned positions and to obtain non-ambiguous sequence alignments to be used in further analyses. A final consensus sequence was generated for each group of samples (n = 28), hereafter identified as *w*AlbA-MID (n = 14) and *w*AlbB-MID (n = 14). These consensus sequences were processed through the Basic Local Alignment Search Tool (BLAST, https://blast.ncbi.nlm.nih.gov/) to identify regions of similarities between biological sequences existing in the GenBank. Sequencing analyses were performed to compare *Wolbachia* sequences in the study group with those nucleotide sequences of *Wolbachia* strains A (n = 78) and B (n = 58) reported for *Ae. albopictus* in the GenBank database.

**Figure 1:**
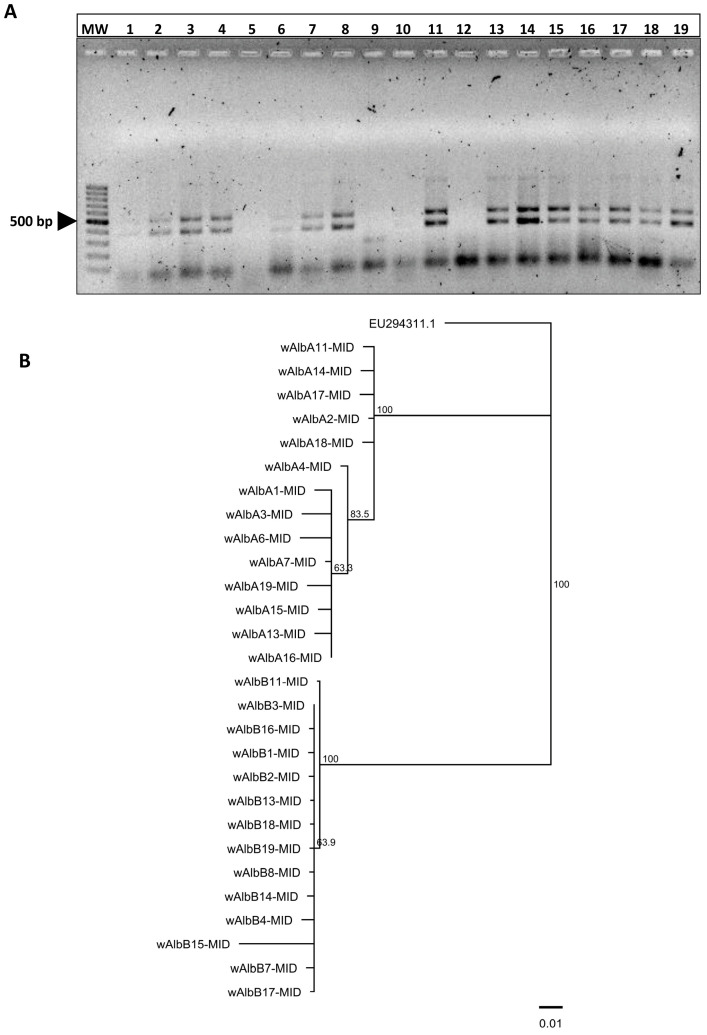
Molecular characterization and nucleotide sequence relationship between *Wolbachia* strains in *Ae. albopictus* of Yucatan. (**A**) PCR amplification and sequencing of a DNA fragment of the *wsp* gene of *Wolbachia* strains A and B in *Ae. albopictus*. Amplicons with lengths of ~300–400 and ~500–600 base pairs (bp) were separated by electrophoresis on agarose gel (1.5%) (lanes 1–19). DNA Marker: 100 bp (lane 1). (**B**) Rooted maximum likelihood phylogenetic tree of the *wsp* gene of 28 *Wolbachia* strains detected in *Ae. albopictus* of Yucatan. Strains are designated as wAlbA-MID (n = 14) and wAlbB-MID (n = 14), which include the initial for *Wolbachia* (w) followed by the abbreviated names of their host species (*Ae. albopictus*: Alb), the *Wolbachia* supergroup that these strains may belong to (A and B), an assigned sample number (1–14), and the collection site (Merida: MID). The tree was inferred using Neighbor-Joining consensus tree Nucleotide alignment (MUSCLE) of all edited *Wolbachia* nucleotide sequences included in the study group and rooted with the *Wolbachia* endosymbiont outer surface protein precursor (*wsp*) gene of the *Ostrinia furnacalis* strain (wfurA) used as an outgroup (accession: EU294311.1). The Jukes–Cantor genetic distance substitution model was used for Bayesian analysis, built using Geneious Tree Builder (Geneious Sofware v6.1.8). The numbers on the branches indicate percentage bootstrap support for major branches obtained from 1000 replicates. Only bootstrap values above 60% that support greedy clustering are shown. The bar indicates a branch length of 0.01.

**Figure 2:**
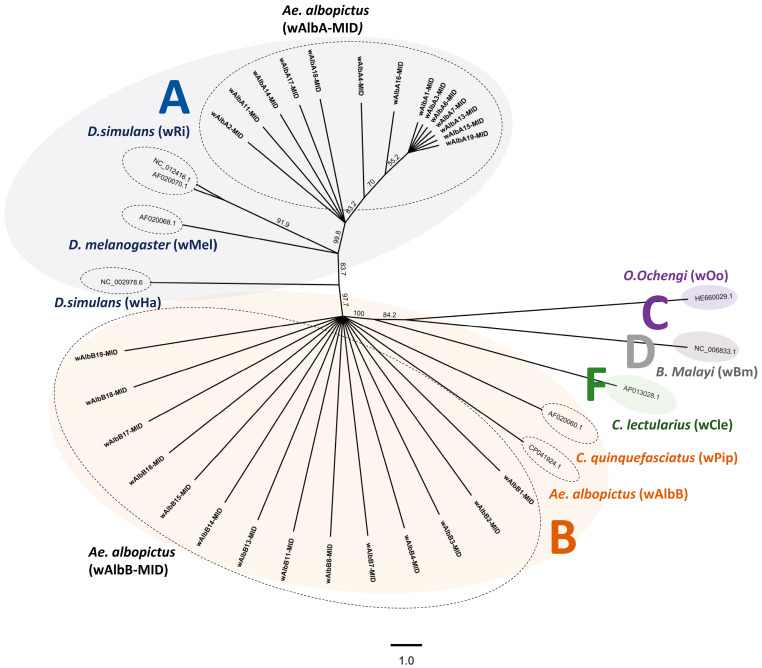
The *Wolbachia* strains in *Aedes albopictus* of Yucatan belong to supergroups A and B. Unrooted maximum likelihood phylogenetic tree showing the relationship between the *wsp* gene of Wolbachia strains detected in *Ae. albopictus* of Yucatan (wAlbA-MID, n = 14; wAlbB- MID, n = 14) (in bold) and distinct *Wolbachia* strains representing different *Wolbachia* supergroups described to infect *Ae. albopictus* elsewhere. *Wolbachia* supergroup affiliations are given in colored circles and indicated by the letter inside the circle next to the host species names as follows: *Drosophila simulants Riverside* (wRi) (accession: AF02070.1., NC_012416.1) and Hawaii (wHa) strains (accession: AF020068.1), and *D. melanogaster* (wMel) strain (accession: NC_002978.6) from supergroup A; *Culex quinquefasciatus* (wPip) (accession: AF020060.1) and *Ae. albopictus* (wAlbB) strains from supergroup B (accession: CP041924.1); *Onchocerca ochengi* (wOo) strain of supergroup C (accession: HE660029.1); *Brugia malayi* (wBm) strain representing supergroup D (accession: NC_006833.1), and *Cimex lectularius* (wCle) strain in supergroup F. Taxon labels correspond to Wolbachia strain names as well as Genbank accession numbers. Nucleotide sequences belonging to the study group as well as their species of origin are depicted in bold. Primarily greedy clustering was used as the consensus method. The numbers on clades correspond to bootstrap values, presented as percentages, from 1000 itinerations, as well as consensus support (%) between clades. The scale bar corresponds to inferred evolutionary changes and indicates a branch length of 1.0. The Jukes–Cantor genetic distance substitution model was used for Bayesian analysis. The tree was inferred using Neighbor-Joining consensus tree Nucleotide alignment (MUSCLE) of all edited *Wolbachia* nucleotide sequences and built using Geneious Tree Builder (Geneious Sofware v6.1.8). The numbers on branches indicate the percentage bootstrap support for major branches obtained from 1000 replicates. Only bootstrap values of 60% or more obtained by the consensus method are shown. The bar indicates a branch length of 0.01.

**Figure 3:**
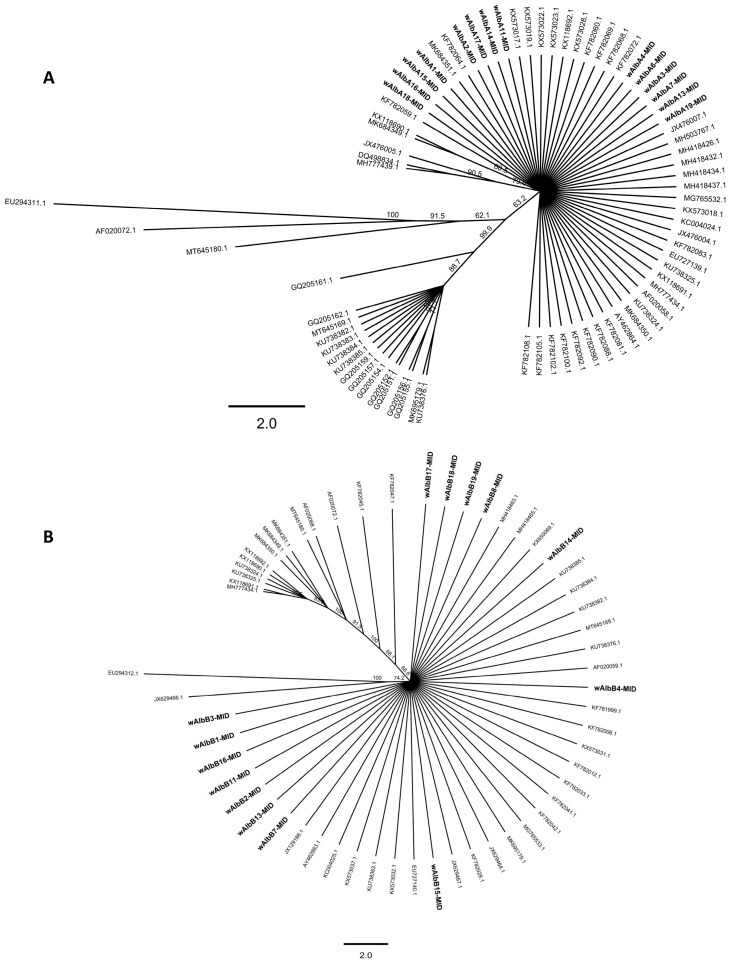
Nucleotide sequence comparison of wAlbA and wAlb-B MID with *Wolbachia* strains belonging to supergroups A and B found in *Ae. albopictus* worldwide. Unrooted phylogenetic tree layout for comparison of *wsp* gene sequences obtained from all *Wolbachia* strains detected in *Ae. albopictus* of Yucatan: (**A**) wAlbA-MID (n = 14) and (**B**) wAlbB-MID (n = 14) (in bold) and other nucleotide sequences of *Wolbachia* strains detected in *Ae. albopictus* reported in GenBank [supergroup A (n = 78); supergroup B (n = 58)]. Strains of the study group are designated as wAlbA-MID or wAlbB-MID, as explained in Figure 1B. The tree was inferred using Neighbor-Joining consensus tree Nucleotide alignment (MUSCLE) of all edited *Wolbachia* nucleotide sequences included in the study group and other *Wolbachia* sequences of *Ae. albopictus* worldwide. The Jukes–Cantor genetic distance substitution model was used for Bayesian analysis, and the phylogeny tree was built using Geneious Tree Builder (Geneious Sofware v6.1.8). The taxon labels correspond to the *Wolbachia* strain names in the study group as well as the GenBank accession numbers. The numbers on branches indicate percentage bootstrap support for major branches obtained from 1000 replicates. Only bootstrap values above 60% or more obtained by the consensus method are shown. The bar indicates a branch length of 2.

Rooted and unrooted maximum likelihood phylogenetic trees of the *wsp* gene were built using Geneious software version 6.1 to show the relationship between the *wsp* gene of representative *Wolbachia* strains detected in *Ae. albopictus* of Yucatán and distinct *Wolbachia* strains representing different supergroups described to infect *Ae. albopictus* worldwide. The Jukes–Cantor genetic distance substitution model was used for Bayesian analysis to infer the evolutionary history of the *Wolbachia* nucleotide sequences included in the study [36]. The bar indicates a branch length of 0.01. Bootstrap values were obtained from 1000 replicates (Figure 1). The GenBank accession numbers for each nucleotide sequence used in the study are included in each figure (Figure 2 and Figure 3). Finally, to provide complementary information to the *wsp* gene DNA sequencing data and allow a more detailed description of the potential intra-strain variability of *Wolbachia* strains A and B in *Ae. albopictus* from Yucatán, we analyzed distinct genetic diversity indices, including haplotype richness (number of haplotypes), haplotype diversity (Hd), nucleotide diversity (π), and mean genetic distance (RStudio. V4.5.1, https://cran.r-project.org/bin/windows/base/ accessed on 1 June 2023).

## 3. Results and Discussion

### 3.1. Detection of Wolbachia Strains A and B in Field-Collected Aedes albopictus from Yucatán

In previous studies, we demonstrated the occurrence of *Wolbachia* infection (~42%, 19/45) in field-caught *Ae. albopictus* collected in the suburban areas of the municipality of Mérida, Yucatán [30]. In this study, 15 out of 19 *Ae. albopictus* specimens showed PCR amplifications for both set of primers specific to the molecular detection of *Wolbachia* strains A and B of *Wolbachia* (molecular weights between 400 and 600 bp) (Figure 1A). These fragments belonged to supergroups A and B (Table 1). The analyzed sequences belonging to the *Wolbachia* strain group A (*w*AlbA-MID) had variable lengths of 185 and 314 nucleotides (n = 14). On the other hand, the length of the sequences belonging to the *Wolbachia* strain group B (*w*AlbB-MID) varied from 252 to 377 nucleotides length (n = 14) (Supplementary Materials).

Furthermore, multiple alignment of several representative nucleotide sequences of the study group [*w*AlbA-MID (n = 4), *w*AlbB-MID (n = 4)] and the nucleotide sequence of the *wsp* gene of the reference strain *Wolbachia pipientis* (accession: AF020070.1) confirmed these similarities and the location of the amplified nucleotide sequences from *Ae. albopictus* of Yucatán within the *wsp* gene of *Wolbachia* (Supplementary Figure S1A,B). Additionally, these results confirmed that *Ae. albopictus* harbors two *Wolbachia* strains from supergroups A and B.

Based on the sequence of the *wsp* gene, other studies have identified high variability between different *Wolbachia* isolates, which can be used to resolve the sequencing relationships of different *Wolbachia* strains [37,38]. Here, we performed a pairwise alignment (MUSCLE) analysis of nucleotide sequences from the *wsp* gene fragments amplified by PCR in *Ae. albopictus* of Yucatán. This analysis identified significant similarities within each study group: the *w*AlbA-MID group (n = 14) showed identity percentages ranging from 98.9 to 100%, and the *w*AlbB-MID group (n = 14) showed identities ranging from 96.9 and 100% (Supplementary Tables S1 and S2). The results of the sequence alignment, as well as consensus sequences for *w*AlbA-MID and *w*AlbB-MID, are presented in Supplementary Figures S2 and S3.

As expected, a comparison between the two groups (*w*AlbA-MID vs. *w*AlbB-MID) revealed limited similarities, ranging from 69.4% to less than 78% (Supplementary Table S3), clearly indicating the presence of two *Wolbachia* strain supergroups in *Ae. albopictus*. Further analysis using the tree builder tool (Geneious software) generated a cladogram that distinctly shows two separated clusters, corresponding to the *w*AlbA-MID group and the *w*AlbB-MID group (Figure 1B).

These results together support and confirm that the *w*AlbA-MID and *w*AlbB-MID strains represent two related (in terms of sequencing) but distinct groups of *Wolbachia* strains circulating in wild-caught *Ae. albopictus* of Yucatán [19,20,22,30,34]. A preliminary analysis of distinct genetic diversity indices revealed that the number of haplotypes (haplotype richness) was higher among *Wolbachia* strain A (7 of 14) compared to strain B (4 of 14), indicating that strain A is richer in variants. In terms of haplotype diversity (Hd), which reflects the probability that two randomly chosen sequences differ, *Wolbachia* strain A showed high diversity (Hd = 0.81), whereas strain B showed moderate diversity (Hd = 0.63). Similarly, nucleotide diversity (π), which measures the average number of nucleotide differences per site between two sequences, was slightly higher in *Wolbachia* strain A (π = 0.0063) compared to strain B (π = 0.0039), indicating that both strains are highly conserved, with strain A being slightly more variable. However, this observed genetic variability within *Wolbachia* strains A and B of *Ae. albopictus* from Yucatan must be interpreted with caution. This study was limited by the collection of mosquitoes from a single location, resulting in a small sample size (n = 45) and a limited number of nucleotide sequences (n = 19). These constraints likely reduced the resolution of intra-population genetic variability. To better understand the extent of *Wolbachia* genetic diversity, future studies should involve more comprehensive field collection. This includes increasing the number of sampled mosquitoes and expanding sampling efforts to include additional urban and suburban locations, ideally with replication across ecological and geographic gradients.

### 3.2. Nucleotide Comparison Analyses with Wolbachia Strains in Aedes albopictus

Consensus tree analyses of the *w*AlbA-MID (n = 14) and *w*AlbB-MID (n = 14) strains, along with reference *Wolbachia* strains from different supergroups (A, B, C, D, and F; see Table 2), revealed distinct phylogenetic clustering. The *w*AlbA-MID strains grouped closely with members of supergroup A, including the *w*Ri and *w*Ha strains previously identified in *Drosophila simulans* [39,40], and *w*Mel identified from *D. melanogaster* [41]. In contrast, *w*AlbB-MID strains clustered with supergroup B strains such as *w*AlbB from *Ae. albopictus* [42], as well as *w*Pip from *Culex quinquefasciatus* [43] (Figure 2). As shown in Figure 2, both groups showed low sequence identity (<35%) with *Wolbachia* strains from unrelated supergroups, including the *w*Cle strain from *Cimex lectularius* (Cimicidae) [44], the *w*Bm strain from *Brugia malayi* (*Filariidae*), and the *w*Oo strain from *Onchocerca ochengi* (*Onchocercidae*) [45,46]. A detailed comparison of representative nucleotide sequence identities, based on alignment analyses, is provided in Supplementary Table S4. These analyses further confirm the distinct phylogenetic identities of the two *Wolbachia* strain groups detected in *Ae. albopictus* of Yucatán.

We also compared the two *w*AlbA-MID and *w*AlbA-MID groups of nucleotide sequences (Figure 1) with *Wolbachia* sequences from supergroups A (n = 78) and B (n = 58) found in *Ae. albopictus* from various geographical regions worldwide (Figure 3A,B). Pairwise multiple sequence alignment revealed that the *w*AlbA-MID group exhibits high sequence identities (>98–100%) with *Wolbachia* strains of supergroup A detected in *Ae. albopictus* from countries including India (JX476004.1, JX476007.1), the USA (e.g., AF020058.1), Malaysia (e.g., KX573028.1, KC004024.1, MH418426.1), Pakistan (MH503767.1), Taiwan (AY462864.1), México (e.g., MK684349.1, KX118691.1), Italy (EU727139.1), China (KU738324.1), and Sri Lanka (MH777434.1) (Figure 3A, Table 3).

Interestingly, lower sequence similarities (<80%) were observed between *Wolbachia* strains A from Brazil (e.g., GQ205162.1), India (e.g., JX476005.1), China (e.g., KU738376.1), México (e.g., MK695179.1), and Singapore (e.g., MT645169.1). These strains were separately grouped into a distinct clade.

For the *w*AlbB-MID group, the multiple alignment analyses revealed high sequence similarity (>98%) when compared *Wolbachia* strain B previously described in various countries, including India (JX629464.1), Malaysia (JX129186.1, KF781999.1), Pakistan (KX650069.1), the USA (MG765533.1), Italy (EU727140.1), Taiwan (AY462863.1), China (KU738369.1), and Singapore (MT645169.1) (Figure 3B, Table 4). In contrast, fewer sequences, such as those reported in Mexico (e.g., KX118691.1, MK684351.1) and China (e.g., KU738324.1), showed less than 90% sequence similarity (<80%) with the *w*AlbB-MID group of Merida.

Overall, our results suggest that most of the *Wolbachia* strains naturally occurring in populations of *Ae. albopictus* of Mérida are very similar to other *Wolbachia* strains belonging to supergroups A and B of *Ae. albopictus* described worldwide. Interesting, nucleotide sequences belonging to the *w*AlbA-MID group showed lower sequence identities (<80%) compared to *Wolbachia* strains previously identified as part of supergroup A in Brazil (e.g., GQ205162.1). In contrast, the results for *Wolbachia* strains in supergroup B were more consistent with previous studies from Asia (Sri Lanka, India, China, Malaysia, and Thailand), which reported that *Ae. albopictus* and, as well as other *Aedes*-related species such as *Ae. Quadrivittatus*, harbored *Wolbachia* strain B with strong bootstrap support [50,51,52,53,54]. Additional genetic markers such as MLST, including additional *Wolbachia* genes (e.g., gatB, coxA, hcpA, ftsZ, and fbpA), will enable more in-depth phylogenetic analyses to better define the genetic diversity among *Wolbachia* strains in *Ae. albopictus* and other *Aedes* species. It is well established that *Ae. albopictus* naturally carries either one strain (*w*AlbA or *w*AlbB) or both strains simultaneously (*w*AlbA and *w*AlbB) [46,50].

Despite this, *Ae. albopictus* continues to expand as an important vector for arbovirus transmission [14,15,16,17]. From a vector control perspective, this poses a challenge for the use of *Ae. albopictus* in *Wolbachia*-based control programs, which aim to suppress mosquito populations or reduce arbovirus infections in humans. Notably, recent evidence shows that *Ae. albopictus* artificially infected with four different *Wolbachia* strains (*w*Mel, *w*MelPop, *w*Ri, and *w*Pip) have established stable lines with diverse CI patterns and reduced vector capacity for arbovirus transmission [55,56]. These findings support the feasibility of applying mass rearing and integrated SIT to control *Ae. albopictus*, as has already been implemented for *Ae. aegypti* worldwide.

## 4. Conclusions

Here, we confirmed the presence of two *Wolbachia* strains (supergroups A and B) in field-collected *Ae. albopictus* from suburban areas of Mérida, Yucatán, through *wsp* gene sequencing. These results align with previous reports of *Ae. albopictus* naturally harboring *Wolbachia* A and B worldwide, showing high sequence homology with strains from Asia and North America, but lower similarity to strains from Brazil, suggesting regional differentiation. Historical records indicate that *Ae. albocpictus* was introduced in the Americas in 1983 via used-tire shipments to the USA, and later detected in Brazil (1986) and Mexico (1988) [57]. The distribution of related and unrelated *Wolbachia* strains across distant regions likely reflects mosquito dispersal through natural migration or human activities, such as trade. These findings suggest historical connectivity among mosquito populations, with phylogenetic analysis offering insights into the biogeographic history of *Ae. albopictus*.

In Merida, *Ae. albopictus* has only recently been identified in urban and periurban areas of this municipality [32], limiting specimen availability for this study. Despite this constraint, our work provides the first description of *Wolbachia* gene sequencing in *Aedes* mosquitos of Yucatán. These results establish a foundation for future research with larger sample sizes and broader collection areas to assess the abundance, distribution, and diversity of *Wolbachia* strains in *Aedes* populations across Yucatán but also Mexico.

Although *wsp* genes are widely used in phylogenetic studies [58], single-gene analyses may be limited by recombination among *Wolbachia* strains [59]. Our complementary nucleotide diversity (π) analyses showed that strain A was slightly more diverse than strain B (π = 0.0063 vs. 0.0039), though both exhibited low variability, consistent with closely related endosymbionts such as *Wolbachia* A and B. Future studies incorporating additional markers (e.g., *gltA*, *groEL*, *ftsZ*) and multilocus sequence typing (MLST) [60,61] will better characterize *Wolbachia* diversity in Yucatán and México.

In conclusion, our results provide baseline evidence for the presence of *Wolbachia* strains A and B in wild *Ae. albopictus* populations of Yucatán. These findings can inform future *Wolbachia*-based intervention strategies, leveraging locally circulating *Wolbachia* strains to reduce mosquito populations and mitigate arboviral transmission in endemic regions.

## Figures and Tables

**Table 1:** Nucleotide sequence similarities found between *Wolbachia* DNA fragments amplified from field-caught *Ae. albopictus* and nucleotide sequences reported in GenBank (NCBI) using the standard nucleotide BLASTN+2.17.0 (https://blast.ncbi.nlm.nih.gov/ accessed on 1 June 2023).

Accession Number	Max Score	Total Score	Percentage Identity	*Wolbachia* Strain (*wsp* Gene)
MK684349.1	494	494	100	A
KY817484.1	494	494	100	A
KX573028.1	494	494	100	A
KY523670.1	494	494	100	A
KJ140127.1	494	494	100	A
KF725078.1	494	494	100	A
JX476002.1	494	494	100	A
KC668278.1	494	494	100	A
HM007832.1	494	494	100	A
MK684351.1	492	492	99	A
MF805776.1	492	492	100	A
KX118690.1	492	492	100	A
KF725079.1	492	492	100	A
JX475999.1	492	492	100	A
AF020058.1	490	490	100	A
KC668284.1	488	488	99.63	A
EU651894.1	481	481	100	A
MK684350.1	475	475	98.88	A
KU738337.1	472	472	100	A
KJ140133.1	466	466	98.13	A
GQ469985.1	466	466	98.5	A
MH418437.1	457	457	100	A
MN307069.1	756	756	100	B
MK695179.1	756	756	100	B
MK695177.1	756	756	100	B
MK695176.1	756	756	100	B
MK695175.1	756	756	100	B
CP041924.1	756	756	100	B
CP041923.1	756	756	100	B
MH418465.1	756	756	100	B
MH418464.1	756	756	100	B
MH418463.1	756	756	100	B

**Table 2:** Representative *Wolbachia* strains belonging to distinct *Wolbachia* supergroups.

Host Organism	Name of Strain	Supergroup	Accession Number	Reference
*D. simulans*	wRi (Riverside)	A	AF020070.1 NC_012416.1	Braig et al., 1998 [35]; Baião et al., 2019 [47]
*D. simulans*	wHa (Hawaii)	A	AF020068.1	Braig et al., 1998 [35]
*D. melanogaster*	wMel	A	NC_002978.6	Wu et al., 2004 [48]
*C. quinquefasciatus*	wPip	B	AF020060.1	Zhou et al., 1998 [3]
*Ae. albopictus*	wAlbB	B	CP041924.1	Kulkarni et al., 2019 [26]
*Onchocerca ochengi*	wOo	C	HE660029.1	Darby et al., 2012 [49]
*Brugia malayi*	wBm	D	NC_006833.1	Foster et al., 2005 [45]
*Cimex lectularius*	wCle	F	AP013028.1	Nikoh et al., 2014 [44]

**Table 3:** *Wolbachia* strain A of *Ae. Albopictus*, with high (98–100%) sequence identity compared to the wAlbA-MID group. GenBank accession numbers and places (country) of detection are shown.

Accession Number	Country
JX476004/7.1	India (Orissa)
AF020058.1 MG765532.1	USA
MH418426/32/34/37.1 KX573017/18/19/22/23/38 KF782059/60/68/72/81/83/88/90 KF782100/02/05/08.1 KC004024.1	Malaysia
MH503767.1	Pakistan
AY462864.1	Taiwan
MK684349/50/51 KX118690/91/92.1	Mexico
EU727139.1	Italy
KU738324/25.1	China
MH777434.1	Sri Lanka

**Table 4:** *Wolbachia* strain B of *Ae. Albopictus*, with high (98–100%) sequence identity compared to the wAlbB-MID group. GenBank accession numbers and places (country) of detection are shown.

Accession Number	Country
JX629464/67	India (Orissa)
JX129186.1 KX5731/32/37.1 MH418463/65.1 KF781999/06/12/28/33/41/42/45/47.1	USA
KX650069.1	Pakistan (Punjab)
MG765533.1 AF020059.1	USA
EU727140.1	Italy
AY462863.1	Taiwan
KU738369/76/82/83/84/85.1	China
MT645169.1	Singapore

## Data Availability

The data presented in this study is included in the article/Supplementary Materials. Further inquiries can be directed to the corresponding authors.

## Supplementary Materials

The following supporting information can be downloaded at: https://www.mdpi.com/article/10.3390/biology14101399/s1, All supplementary material has been submitted along with the main manuscript. Figure S1. (A) Multiple nucleotide sequence alignment between representative wAlbA- MID (n = 4) and wAlbB-MID (n = 4) sequences and the *wsp* gene sequence of a reference Wolbachia strain (accession AF020070.1) (MUSCLE alignment: Geneious software version 6.1). Maximum number of iterations: 10. Agreement between DNA nucleotide sequences are shown in bright colors: adenine (A, red), thymine (T, green), cytosine (C, blue), and guanine (G, yellow). Strains are designated as wAlbA- MID and wAlbB-MID which includes the initial for Wolbachia (w) followed by the abbreviated name of their host species (*Ae. albopictus*: Alb), the Wolbachia supergroup that these strains may belong to (A and B), a sample designed number (1–14), and the collection site (Merida: MID). (B) Schematic representation of Wolbachia complete DNA genome depicting the nucleotide sequence location of the gene encoding for the outer membrane protein precursor (*wsp*) (~779 bp) (accession: AF020070). Figure S2. Multiple nucleotide sequence alignment view of the Wolbachia nucleotide sequences amplified from *Ae. albopictus* of Yucatan. A total of 28 DNA sequences corresponding to the *wsp* gene of the Wolbachia endosymbionts detected in *Ae. albopictus* of Yucatan were separately analyzed through the multiple alignment tool (MUSCLE) of the Geneious software version 6.1. Strains in the study group are designated as (A) wAlbA-MID and (B) wAlbB-MID which include the initial for Wolbachia (w) followed by the abbreviated name of their host species (*Ae. albopictus*: Alb), the Wolbachia supergroup that these strains may belong to (A and B), a sample designed number (1–14), and the collection site (Merida: MID). Nucleotide sequence length is indicated by the number above the set of sequences. Wolbachia endosymbiont outer surface protein precursor (wsp) gene of *Ostrinia furnacalis* strain (wfurA) was used as an outgroup control (accession: EU294311.1). Nucleotide alignment (MUSCLE) of all edited Wolbachia nucleotide sequences was performed using the Neighbor-joining consensus method. Maximum number of iterations used: 10. DNA nucleotide agreement between all sequences are shown in bright colors: adenine (A, red), thymine (T, green), cytosine (C, blue), and guanine (G, yellow). Agreements inside each study group are shown in gray. Bases matching at least 99% of the sequences. The percentage of sequence coverage and identity as well as the generated consensus sequence after multiple alignment analyses are shown at the top as indicated in the figure. Figure S3. Multiple nucleotide sequence alignment view of the Wolbachia nucleotide sequences amplified from *Ae. albopictus* of Yucatan. A total of 28 DNA sequences corresponding to the *wsp* gene of the Wolbachia endosymbionts detected in *Ae. albopictus* of Yucatan were analyzed through the multiple alignment tool (MUSCLE) of the Geneious software version 6.1. Strains in the study group are designated as (A) wAlbA-MID and (B) wAlbB-MID which include the initial for Wolbachia (w) followed by the abbreviated name of 568 their host species (*Ae. albopictus*: Alb), the Wolbachia supergroup that these strains may belong to (A and B), a sample designed number (1–14), and the collection site (Merida: MID). Nucleotide sequence length is indicated by the number above the set of sequences. Wolbachia endosymbiont outer surface protein precursor (*wsp*) gene of *Ostrinia furnacalis* strain (wfurA) was used as an outgroup control (accession: EU294311.1). Nucleotide alignment (MUSCLE) of all edited Wolbachia nucleotide sequences was performed using the Neighbor-joining consensus method. Maximum number of iterations used: 10. DNA nucleotide agreement between all sequences are shown in bright colors: adenine (A, red), thymine (T, green), cytosine (C, blue), and guanine (G, yellow). Agreements inside each study group are shown in gray. Bases matching at least 99% of the sequences. A small view of the phylogenetic organization of these sequences into a tree format is shown in blue (left side). Supplementary Table S1. Pairwise sequence comparison of the nucleotide sequences of wAlbA-MID strains (n = 14) detected in *Aedes albopictus* of Yucatan. Table S2. Pairwise sequence comparison of the nucleotide sequences of wAlbB-MID strains (n = 14) detected in *Aedes albopictus* of Yucatan. Table S3. Heatmap of the nucleotide sequence identity similarities (%) between all wAlbA-MID and wAlbB-MID strains found in *Ae. albopictus* of Yucatan. Table S4. Pairwise sequence comparison of representative nucleotide sequences of the wAlb-MID strains (n = 8) detected in *Aedes albopictus* of Yucatan and reference sequences belonging to multiple *Wolbachia* serogroups.

## Abbreviations

The following abbreviations are used in this manuscript:

*w*AlbA	*Wolbachia* strain A of *Ae. albopictus*.
*w*AlbB	*Wolbachia* strain B of *Ae. albopictus*.
*wsp*	*Wolbachia* surface protein
MID	Mérida
ABVs	*Aedes*-borne human viruses
DENV	Dengue virus
ZIKV	Zika virus
CHIKV	Chikungunya virus
ABD	*Aedes*-borne human diseases
