# Supplementary material for: Wolbachia Surface Protein (wsp) Gene Sequencing of Strains A and B in Native Aedes albopictus of Mérida, Yucatán
Source: Biology (Basel). 2025 Oct 13;14(10):1399. doi: 10.3390/biology14101399 (PMC12561236; doi:10.3390/biology14101399)
Supplement: Supplementary file 1 [file biology-14-01399-s001.zip › biology-3790890-supplementary.pdf]

Supplementary Figure 1

A

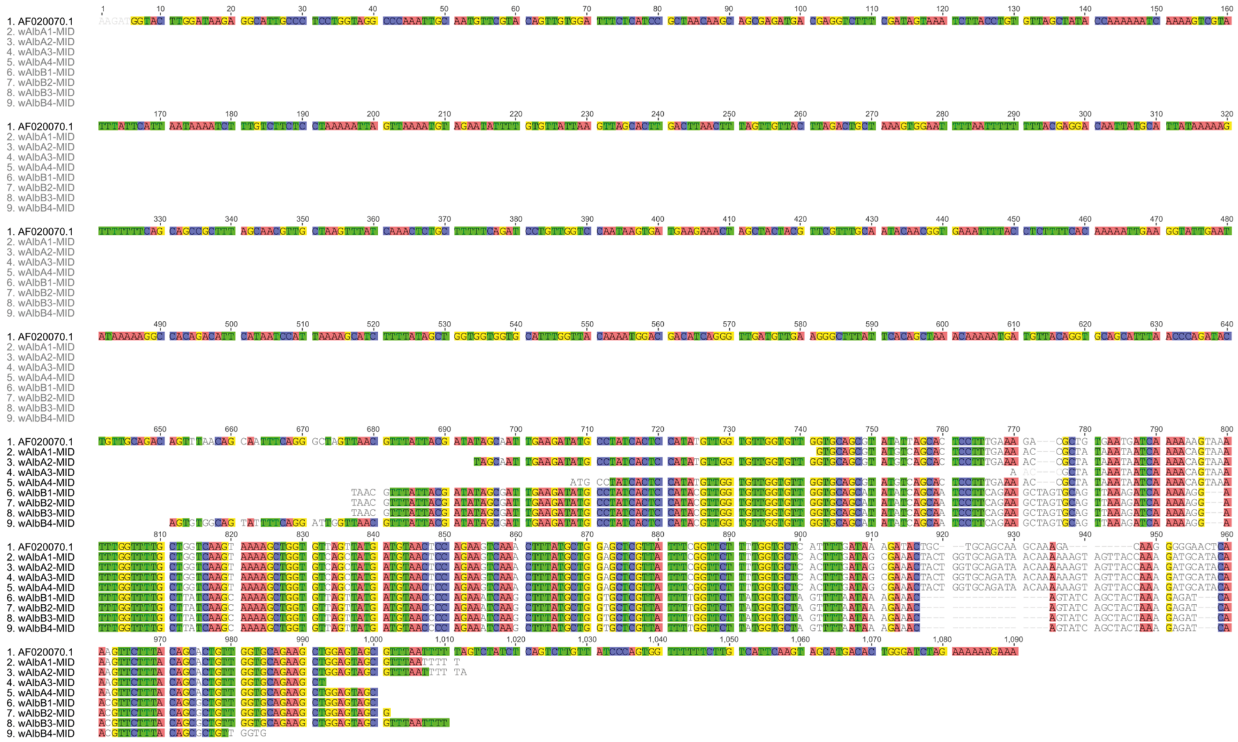

B

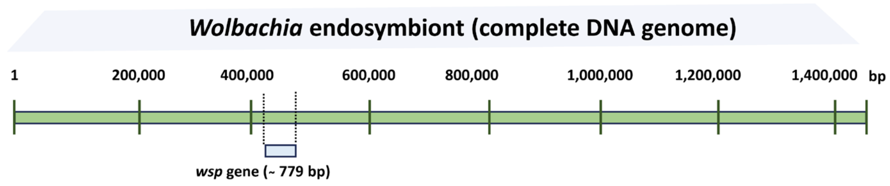

Supplementary Figure S1. (A) Multiple nucleotide sequence alignment between representative wAlbA-MID (n=4) and wAlbB-MID (n=4) sequences and the *wsp* gene sequence of a reference Wolbachia strain (accession AF020070.1) (MUSCLE alignment: Geneious software version 6.1). Maximum number of iterations: 10. Agreement between DNA nucleotide sequences are shown in bright colors: adenine (A, red), thymine (T, green), cytosine (C, blue), and guanine (G, yellow). Strains are designated as wAlbA-MID and wAlbB-MID which includes the initial for Wolbachia (w) followed by the abbreviated name of their host species (*Ae. albopictus*: Alb), the Wolbachia supergroup that these strains may belong to (A and B), a sample designed number (1-14), and the collection site (Merida: MID). (B) Schematic representation of Wolbachia complete DNA genome depicting the nucleotide sequence location of the gene encoding for the outer membrane protein precursor (*wsp*) (~ 779 bp) (accession: AF020070).

Supplementary Figure 2

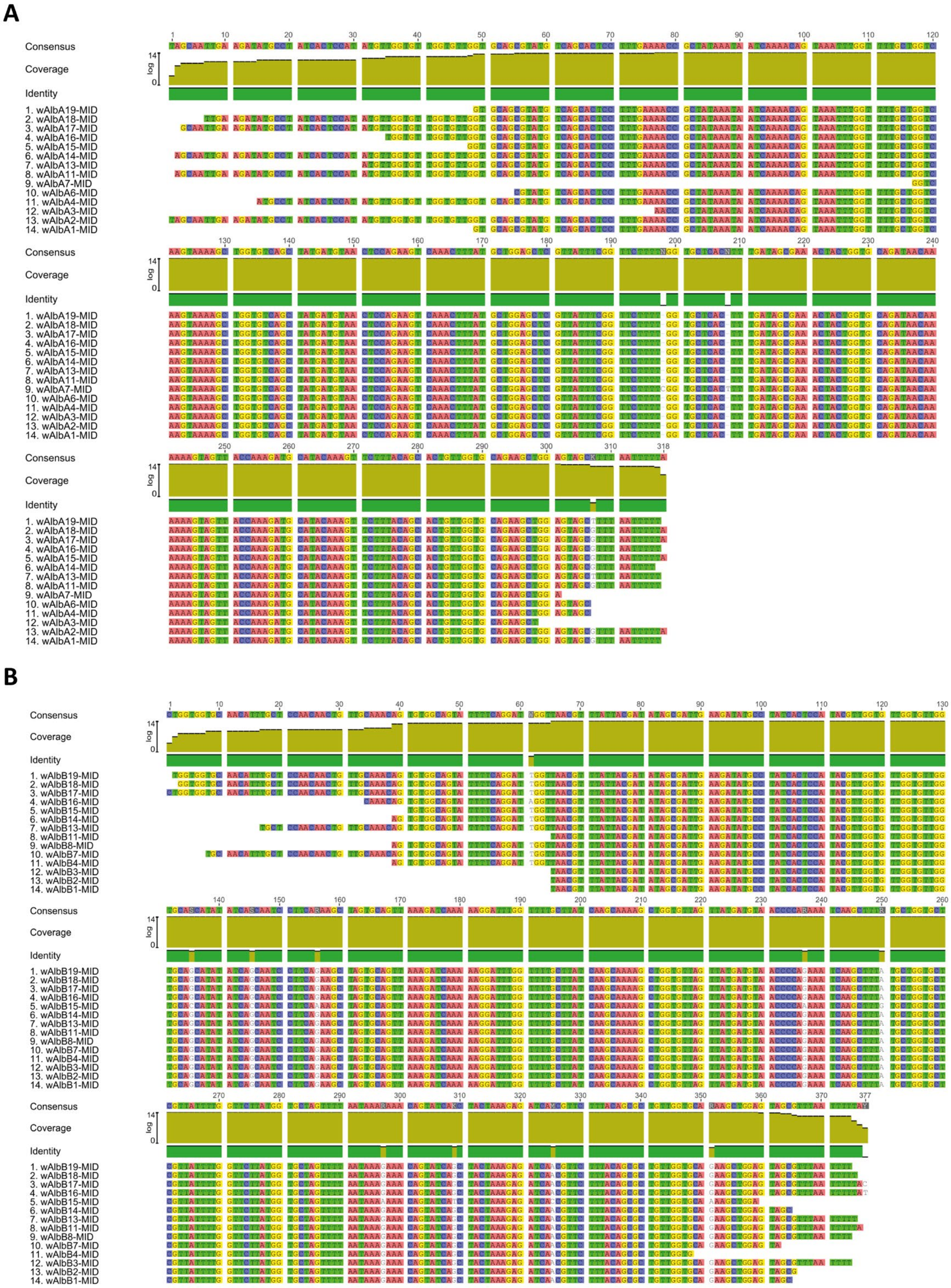

Supplementary Figure S2. Multiple nucleotide sequence alignment view of the Wolbachia nucleotide sequences amplified from *Ae. albopictus* of Yucatan. A total of 28 DNA sequences corresponding to the *wsp* gene of the Wolbachia endosymbionts detected in *Ae. albopictus* of Yucatan were separately analyzed through the multiple alignment tool (MUSCLE) of the Geneious software version 6.1. Strains in the study group are designated as (A) wAlbA-MID and (B) wAlbB-MID which include the initial for Wolbachia (w) followed by the abbreviated name of their host species (*Ae. albopictus*: Alb), the Wolbachia supergroup that

these strains may belong to (A and B), a sample designed number (1-14), and the collection site (Merida: MID). Nucleotide sequence length is indicated by the number above the set of sequences. Wolbachia endosymbiont outer surface protein precursor (wsp) gene of *Ostrinia furnacalis* strain (wfurA) was used as an outgroup control (accession: EU294311.1). Nucleotide alignment (MUSCLE) of all edited Wolbachia nucleotide sequences was performed using the Neighbor-joining consensus method. Maximum number of iterations used: 10. DNA nucleotide agreement between all sequences are shown in bright colors: adenine (A, red), thymine (T, green), cytosine (C, blue), and guanine (G, yellow). Agreements inside each study group are shown in gray. Bases matching at least 99% of the sequences. The percentage of sequence coverage and identity as well as the generated consensus sequence after multiple alignment analyses are shown at the top as indicated in the figure.

Supplementary Figure 3

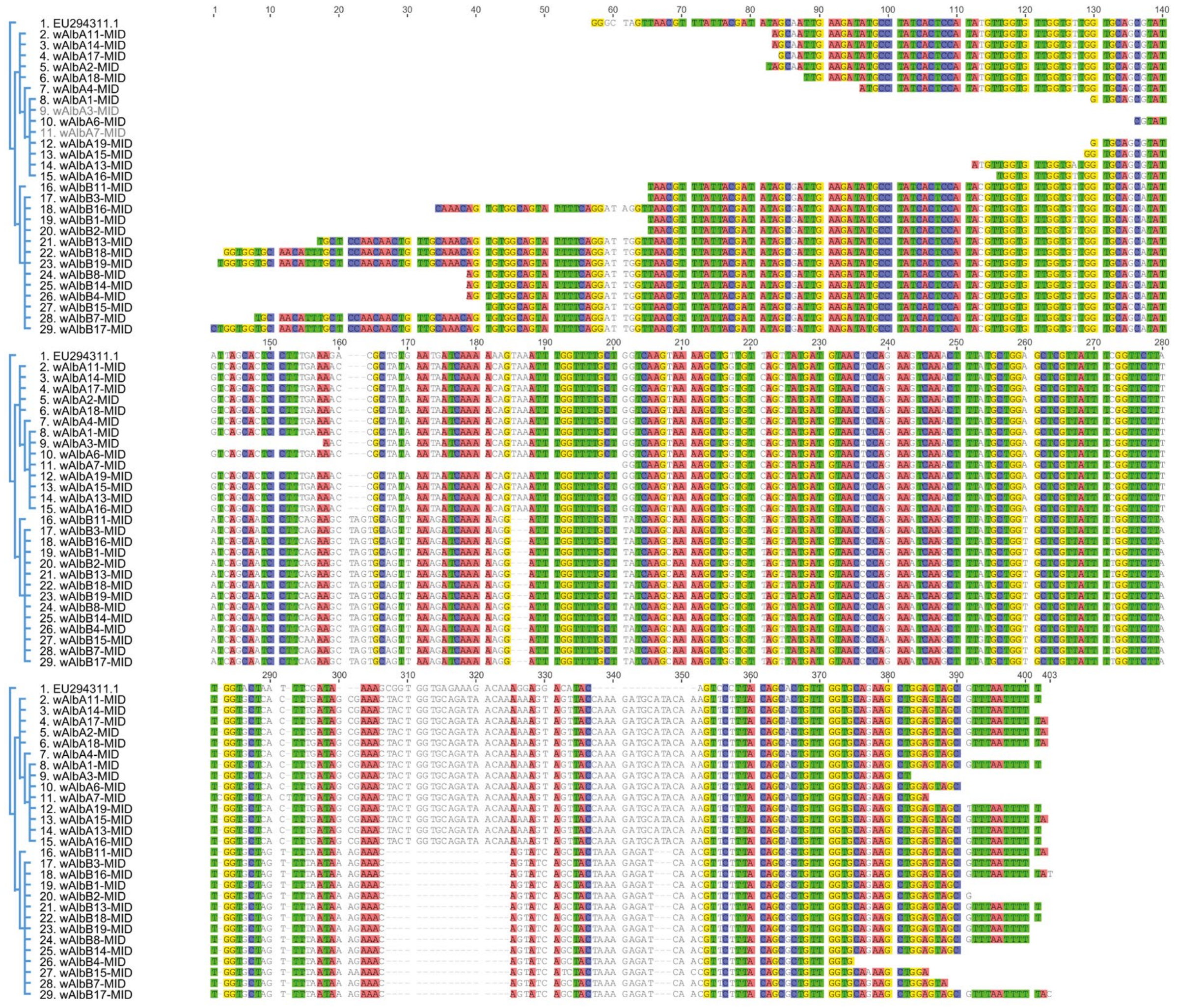

Supplementary Figure S3. Multiple nucleotide sequence alignment view of the Wolbachia nucleotide sequences amplified from *Ae. albopictus* of Yucatan. A total of 28 DNA sequences corresponding to the *wsp* gene of the Wolbachia endosymbionts detected in *Ae. albopictus* of Yucatan were analyzed through the multiple alignment tool (MUSCLE) of the Geneious software version 6.1. Strains in the study group are designated as (A) wAlbA-MID and (B) wAlbB-MID which include the initial for Wolbachia (w) followed by the abbreviated name of 568 their host species (*Ae. albopictus*: Alb), the Wolbachia supergroup that these strains may belong to (A and B), a sample designed number (1-14), and the collection site (Merida: MID). Nucleotide sequence length is indicated by the number above the set of sequences. Wolbachia endosymbiont outer surface protein precursor (*wsp*) gene of *Ostrinia furnacalis* strain (wfurA) was used as an outgroup control (accession: EU294311.1). Nucleotide alignment (MUSCLE) of all edited Wolbachia nucleotide sequences was performed using the Neighbor-joining consensus method. Maximum number of iterations used: 10. DNA nucleotide agreement between all sequences are shown in bright colors: adenine

(A, red), thymine (T, green), cytosine (C, blue), and guanine (G, yellow). Agreements inside each study group are shown in gray. Bases matching at least 99% of the sequences. A small view of the phylogenetic organization of these sequences into a tree format is shown in blue (left side).

Supplementary Table S1. Pairwise sequence comparison of the nucleotide sequences of wAlbA-MID strains (n=14) detected in *Aedes albopictus* of Yucatan.

	wAlbA19-MID	wAlbA18-MID	wAlbA17-MID	wAlbA16-MID	wAlbA15-MID	wAlbA14-MID	wAlbA13-MID	wAlbA11-MID	wAlbA7-MID	wAlbA6-MID	wAlbA4-MID	wAlbA3-MID	wAlbA2-MID	wAlbA1-MID
wAlbA19-MID		99.6	99.6	99.6	99.6	99.6	100	99.6	98.9	100	100	100	99.6	99.6
wAlbA18-MID	99.6		100	100	100	100	99.6	100	98.9	100	100	100	100	100
wAlbA17-MID	99.6	100		100	100	100	99.6	100	98.9	100	100	100	100	100
wAlbA16-MID	99.6	100	100		100	100	99.6	100	98.9	100	100	100	100	100
wAlbA15-MID	99.6	100	100	100		100	99.6	100	98.9	100	100	100	100	100
wAlbA14-MID	99.6	100	100	100	100		99.6	100	98.9	100	100	100	100	100
wAlbA13-MID	100	99.6	99.6	99.6	99.6	99.6		99.6	98.9	100	100	100	99.6	99.6
wAlbA11-MID	99.6	100	100	100	100	100	99.6		98.9	100	100	100	100	100
wAlbA7-MID	98.9	98.9	98.9	98.9	98.9	98.9	98.9	98.9		98.9	98.9	98.9	98.9	98.9
wAlbA6-MID	100	100	100	100	100	100	100	100	98.9		100	100	100	100
wAlbA4-MID	100	100	100	100	100	100	100	100	98.9	100		100	100	100
wAlbA3-MID	100	100	100	100	100	100	100	100	98.9	100	100		100	100
wAlbA2-MID	99.6	100	100	100	100	100	99.6	100	98.9	100	100	100		100
wAlbA1-MID	99.6	100	100	100	100	100	99.6	100	98.9	100	100	100	100	

Each value represents the percentage (%) of the nucleotide sequence identity determined for each *Wolbachia* strain after nucleotide alignment using the MUSCLE tool (Geneious software version 6.1).

Supplementary Table S2. Pairwise sequence comparison of the nucleotide sequences of wAlbB-MID strains (n=14) detected in *Aedes albopictus* of Yucatan.

	wAlbB19-MID	wAlbB18-MID	wAlbB17-MID	wAlbB16-MID	wAlbB15-MID	wAlbB14-MID	wAlbB13-MID	wAlbB11-MID	wAlbB8-MID	wAlbB7-MID	wAlbB4-MID	wAlbB3-MID	wAlbB2-MID	wAlbB1-MID
wAlbB19-MID		100	100	99.7	97.2	100	100	100	100	100	100	100	100	100
wAlbB18-MID	100		100	99.7	97.2	100	100	100	100	100	100	100	100	100
wAlbB17-MID	100	100		99.4	97.2	100	100	100	100	100	100	100	100	100
wAlbB16-MID	99.7	99.7	99.4		96.9	99.7	99.7	100	99.7	99.7	99.7	100	100	100
wAlbB15-MID	97.2	97.2	97.2	96.9		97.2	97.2	96.9	97.2	97.2	97.4	96.9	96.9	96.9
wAlbB14-MID	100	100	100	99.7	97.2		100	100	100	100	100	100	100	100
wAlbB13-MID	100	100	100	99.7	97.2	100		100	100	100	100	100	100	100
wAlbB11-MID	100	100	100	100	96.9	100	100		100	100	100	100	100	100
wAealbB8-MID	100	100	100	99.7	97.2	100	100	100		100	100	100	100	100
wAlbB7-MID	100	100	100	99.7	97.2	100	100	100	100		100	100	100	100
wAlbB4-MID	100	100	100	99.7	97.4	100	100	100	100	100		100	100	100
wAlbB3-MID	100	100	100	100	96.9	100	100	100	100	100	100		100	100
wAlbB2-MID	100	100	100	100	96.9	100	100	100	100	100	100	100		100
wAlbB1-MID	100	100	100	100	96.9	100	100	100	100	100	100	100	100	

Each value represents the percentage (%) of the nucleotide sequence identity determined for each *Wolbachia* strain after nucleotide alignment using the MUSCLE tool (Geneious software version 6.1).

Supplementary Table S3. Heatmap of the nucleotide sequence identity similarities (%) between all wAlbA-MID and wAlbB-MID strains found in *Ae. albopictus* of Yucatan.

	A16-MID	A11-MID	A2-MID	A1-MID	A4-MID	A14-MID	A15-MID	A17-MID	A18-MID	A7-MID	A19-MID	A13-MID	A3-MID	A6-MID	B15-MID	B16-MID	B4-MID	B7-MID	B19-MID	B14-MID	B11-MID	B1-MID	B2-MID	B3-MID	B8-MID	B13-MID	B17-MID	B18-MID
A16-MID		100	100	100	100	100	100	100	100	98.4	99.6	99.3	100	100	71.3	75.4	72.8	74.2	75.3	74.4	75.4	74.4	74.5	75.3	75.3	75.4	75.4	75.4
A11-MID	100		100	100	100	100	100	100	100	98.4	99.6	99.3	100	100	73.8	77.3	75.2	76.3	77.2	76.5	77.3	76.5	76.5	77.2	77.2	77.3	77.3	77.3
A2-MID	100	100		100	100	100	100	100	100	98.4	99.6	99.3	100	100	73.8	77.4	75.3	76.4	77.3	76.5	77.4	76.5	76.6	77.3	77.3	77.4	77.4	77.4
A1-MID	100	100	100		100	100	100	100	100	98.4	99.6	99.6	100	100	69.7	74.1	71.2	72.8	74	73	74.1	73	73.1	74	74	74.1	74.1	74.1
A4-MID	100	100	100	100		100	100	100	100	98.4	100	99.6	100	100	72.9	75.8	74.4	75.6	75.8	75.8	75.8	75.8	75.8	75.8	75.8	75.8	75.8	75.8
A14-MID	100	100	100	100	100		100	100	100	98.4	99.6	99.3	100	100	73.8	77.2	75.2	76.3	77.2	76.5	77.2	76.5	76.5	77.2	77.2	77.2	77.2	77.2
A15-MID	100	100	100	100	100	100		100	100	98.4	99.6	99.6	100	100	69.8	74.3	71.3	72.9	74.1	73.1	74.3	73.1	73.2	74.1	74.1	74.2	74.3	74.2
A17-MID	100	100	100	100	100	100	100		100	98.4	99.6	99.3	100	100	73.7	77.3	75.1	76.2	77.1	76.4	77.3	76.4	76.5	77.1	77.1	77.2	77.3	77.2
A18-MID	100	100	100	100	100	100	100	100		98.4	99.6	99.3	100	100	73.6	77.3	75.1	76.3	77.2	76.4	77.3	76.4	76.5	77.2	77.2	77.2	77.3	77.2
A7-MID	98.4	98.4	98.4	98.4	98.4	98.4	98.4	98.4	98.4		98.4	98.4	98.4	98.4	69.7	73	71.3	73	73	73	73	73	73	73	73	73	73	73
A19-MID	99.6	99.6	99.6	99.6	100	99.6	99.6	99.6	99.6	98.4		100	100	100	69.7	73.7	71.2	72.8	73.6	73	73.7	73	72.7	73.6	73.6	73.7	73.7	73.7
A13-MID	99.3	99.3	99.3	99.6	99.6	99.3	99.6	99.3	99.3	98.4	100		100	100	71	74.7	72.4	73.8	74.6	74	74.7	74	73.7	74.6	74.6	74.7	74.7	74.7
A3-MID	100	100	100	100	100	100	100	100	100	98.4	100	100		100	68.6	71.3	70.2	71.3	71.3	71.3	71.3	71.3	71.3	71.3	71.3	71.3	71.3	71.3

**A6-MID**	100	100	100	100	100	100	100	100	100	98.4	100	100	100		69.4	72.3	70.5	72.1	72.3	72.3	72.3	72.3	72.3	72.3	72.3	72.3	72.3	72.3
**B15-MID**	71.3	73.8	73.8	69.7	72.9	73.8	69.8	73.7	73.6	69.7	69.7	71	68.6	69.4		96.9	97.4	97.2	97.2	97.2	96.9	96.9	96.9	96.9	97.2	97.2	97.2	97.2
**B16-MID**	75.4	77.3	77.4	74.1	75.8	77.2	74.3	77.3	77.3	73	73.7	74.7	71.3	72.3	96.9		99.7	99.7	99.7	99.7	100	100	100	100	99.7	99.7	99.4	99.7
**B4-MID**	72.8	75.2	75.3	71.2	74.4	75.2	71.3	75.1	75.1	71.3	71.2	72.4	70.2	70.5	97.4	99.7		100	100	100	100	100	100	100	100	100	100	100
**B7-MID**	74.2	76.3	76.4	72.8	75.6	76.3	72.9	76.2	76.3	73	72.8	73.8	71.3	72.1	97.2	99.7	100		100	100	100	100	100	100	100	100	100	100
**B19-MID**	75.3	77.2	77.3	74	75.8	77.2	74.1	77.1	77.2	73	73.6	74.6	71.3	72.3	97.2	99.7	100	100		100	100	100	100	100	100	100	100	100
**B14-MID**	74.4	76.5	76.5	73	75.8	76.5	73.1	76.4	76.4	73	73	74	71.3	72.3	97.2	99.7	100	100	100		100	100	100	100	100	100	100	100
**B11-MID**	75.4	77.3	77.4	74.1	75.8	77.2	74.3	77.3	77.3	73	73.7	74.7	71.3	72.3	96.9	100	100	100	100	100		100	100	100	100	100	100	100
**B1-MID**	74.4	76.5	76.5	73	75.8	76.5	73.1	76.4	76.4	73	73	74	71.3	72.3	96.9	100	100	100	100	100	100		100	100	100	100	100	100
**B2-MID**	74.5	76.5	76.6	73.1	75.8	76.5	73.2	76.5	76.5	73	72.7	73.7	71.3	72.3	96.9	100	100	100	100	100	100	100		100	100	100	100	100
**B3-MID**	75.3	77.2	77.3	74	75.8	77.2	74.1	77.1	77.2	73	73.6	74.6	71.3	72.3	96.9	100	100	100	100	100	100	100	100		100	100	100	100
**B8-MID**	75.3	77.2	77.3	74	75.8	77.2	74.1	77.1	77.2	73	73.6	74.6	71.3	72.3	97.2	99.7	100	100	100	100	100	100	100	100		100	100	100
**B13-MID**	75.4	77.3	77.4	74.1	75.8	77.2	74.2	77.2	77.2	73	73.7	74.7	71.3	72.3	97.2	99.7	100	100	100	100	100	100	100	100	100		100	100
**B17-MID**	75.4	77.3	77.4	74.1	75.8	77.2	74.3	77.3	77.3	73	73.7	74.7	71.3	72.3	97.2	99.4	100	100	100	100	100	100	100	100	100	100		100
**B18-MID**	75.4	77.3	77.4	74.1	75.8	77.2	74.2	77.2	77.2	73	73.7	74.7	71.3	72.3	97.2	99.7	100	100	100	100	100	100	100	100	100	100	100	

wAlbA-MID and wAlbB-MID strains are here identified as A-MID and B-MID, respectively.

Supplementary Table S4. Pairwise sequence comparison of representative nucleotide sequences of the wAlb-MID strains (n=8) detected in *Aedes albopictus* of Yucatan and reference sequences belonging to multiple *Wolbachia* serogroups.

	**AF020060.1**	**AF020068.1**	**AF020070.1**	**AP013028.1**	**CP041924.1**	**HE660029.1**	**NC_002978.6**	**NC_006833.1**	**NC_012416.1**	**A1-MID**	**A2-MID**	**A3-MID**	**A4-MID**	**B1-MID**	**B2-MID**	**B3-MID**	**B4-MID**
**AF020060.1**		73.4	81.5	30.6	97.5	31.8	76.1	34.2	81.5	69.8	74	68.7	73.1	97.6	97.6	97.6	97.4
**AF020068.1**	73.4		88.7	30.1	75.6	28.1	82.1	30.2	88.8	82.4	85.1	81.4	84.1	78.4	78.5	78.8	77.5
**AF020070.1**	81.5	88.7		31.5	80.8	28.7	88.9	32.1	99.9	86.5	88.6	84.5	87.6	81.9	82	82.5	80
**AP013028.1**	30.6	30.1	31.5		36.7	62.3	32.1	62.3	31.5	35.3	35.3	35.1	34.7	35.6	35.5	35.7	35
**CP041924.1**	97.5	75.6	80.8	36.7		36.9	76.6	37.2	80.8	72.2	75.9	70.4	75.1	100	100	100	100
**HE660029.1**	31.8	28.1	28.7	62.3	36.9		29.1	76.1	28.5	30.7	32.1	30.4	31.3	35.7	35.6	35.8	35.6
**NC_002978.6**	76.1	82.1	88.9	32.1	76.6	29.1		32.8	89	83.5	85.4	81.9	84.5	76.7	76.7	77.1	75.9
**NC_006833.1**	34.2	30.2	32.1	62.3	37.2	76.1	32.8		32.1	33.1	34.3	32.4	33.4	36	35.9	36.1	36.5
**NC_012416.1**	81.5	88.8	99.9	31.5	80.8	28.5	89	32.1		86.5	88.6	84.5	87.6	81.9	82	82.5	80

**A1-MID**	69.8	82.4	86.5	35.3	72.2	30.7	83.5	33.1	86.5		100	100	100	72.2	72.3	73.2	70.4
**A2-MID**	74	85.1	88.6	35.3	75.9	32.1	85.4	34.3	88.6	100		100	100	75.9	76	76.7	74.6
**A3-MID**	68.7	81.4	84.5	35.1	70.4	30.4	81.9	32.4	84.5	100	100		100	70.4	70.4	70.4	69.3
**A4-MID**	73.1	84.1	87.6	34.7	75.1	31.3	84.5	33.4	87.6	100	100	100		75.1	75.1	75.1	73.6
**B1-MID**	97.6	78.4	81.9	35.6	100	35.7	76.7	36	81.9	72.2	75.9	70.4	75.1		100	100	100
**B2-MID**	97.6	78.5	82	35.5	100	35.6	76.7	35.9	82	72.3	76	70.4	75.1	100		100	100
**B3-MID**	97.6	78.8	82.5	35.7	100	35.8	77.1	36.1	82.5	73.2	76.7	70.4	75.1	100	100		100
**B4-MID**	97.4	77.5	80	35	100	35.6	75.9	36.5	80	70.4	74.6	69.3	73.6	100	100	100	

Each value represents the percentage (%) of the nucleotide sequence identity determined for each *Wolbachia* strain after nucleotide alignment using the MUSCLE tool (Geneious software version 6.1). wAlbA-MID (n=3) and wAlbB-MID (n=3) strains are here identified as A-MID and B-MID, respectively. Accession numbers for reference sequences are indicated.
